# Gabapentin, a human therapeutic medication and an environmental substance transferring at trace levels to horses: a case report

**DOI:** 10.1186/s13620-022-00226-5

**Published:** 2022-10-04

**Authors:** Kimberly Brewer, Jacob Machin, George Maylin, Clara Fenger, Abelardo Morales-Briceño, Thomas Tobin

**Affiliations:** 1 Wellington, FL USA; 2 The Maxwell H. Gluck Equine Research Center and Department of Toxicology and Cancer Biology, University of Kentucky, Lexington, Kentucky 40546 USA; 3 New York Drug Testing and Research Program, 777 Warren Rd, Ithaca, NY 14853 USA; 4 Equine Integrated Medicine, 4904 Ironworks Rd., Georgetown, KY 40324 USA; 5 Emirates Endurance Village, Stable 28, Alain Stud-Al Wathba, Abu Dhabi, United Arab Emirates

**Keywords:** Gabapentin, Environmental presence, Horses, Plasma concentration, Screening Limit of Detection, 8 ng/mL

## Abstract

Gabapentin, 1-(Aminomethyl)cyclohexaneacetic acid, MW 171.240, is a frequently prescribed high dose human medication that is also used recreationally. Gabapentin is orally absorbed; the dose can be 3,000 mg/day and it is excreted essentially unchanged in urine. Gabapentin is stable in the environment and routinely detected in urban wastewater. Gabapentin randomly transfers from humans to racing horses and is at times detected at pharmacologically ineffective / trace level concentrations in equine plasma and urine. In Ohio racing between January 2019 and July 2020,18 Gabapentin identifications, all less than 2 ng/ml in plasma, were reported. These identifications were ongoing because the horsemen involved were unable to pin down and therefore avoid the source of these identifications. Given that 44 ng/ml or less is an Irrelevant Plasma Concentration (IPC) of Gabapentin in horses, we proposed a 5 ng/ml plasma interim Screening Limit of Detection for Gabapentin identifications in Ohio racing, and an essentially similar 8 ng/ml plasma Screening Limit of Detection was suggested by a scientific advisor to the Ohio Horse Racing Commission. As such, an analytical Screening Limit of 8 ng /ml in plasma is an appropriate and pharmacologically conservative analytical “cut-off” or Screening Limit of Detection (SLOD) for Gabapentin in equine competitive events to avoid the calling of *“positive”* identifications on random unavoidable trace level identifications of this widely prescribed human therapeutic medication in equine forensic samples.

## Background

Gabapentin, 1-(Aminomethyl)cyclohexaneacetic acid, MW 171.240, is the 11^th^ most frequently prescribed human medication and Gabapentin is also available and used recreationally in the United States. This review starts with the matter of a Standardbred racehorse shipped from Ontario, Canada, to Scioto Downs, Ohio, racing on September 7^th^, 2019. The horse won its race and post-race blood, and urine samples were collected and sent to the Analytical Toxicology Laboratory of the Ohio Department of Agriculture (ODA). The primary “A” post-race blood sample was subjected to a “*preliminary analysis*” which “*seemed to indicate the suspected presence of Gabapentin”,* which “*suspected presence*” was then confirmed [1]. The B split sample analysis confirmed the Ohio laboratory identification of Gabapentin and reported the serum concentration as 273 pg/ml [2]. The connections of the horse /responsible persons were completely unaware of any possible exposure of this horse to Gabapentin. Furthermore, this low concentration plasma Gabapentin *“positive”* was consistent with the year 2018 and thereafter experience of Ohio Harness Horsemen, who were presented with a sequence of about twenty or more low plasma concentration Gabapentin “*positives”*, starting in 2018 and apparently ending in 2020. These low concentration Gabapentin “*positives”* present as a classic series of innocent trace level identification *“positives”* resulting from random inadvertent exposure of these horses to environmental Gabapentin, as we will now detail.

## The ongoing gabapentin “positives” in Ohio racing

**Fig. 1:**
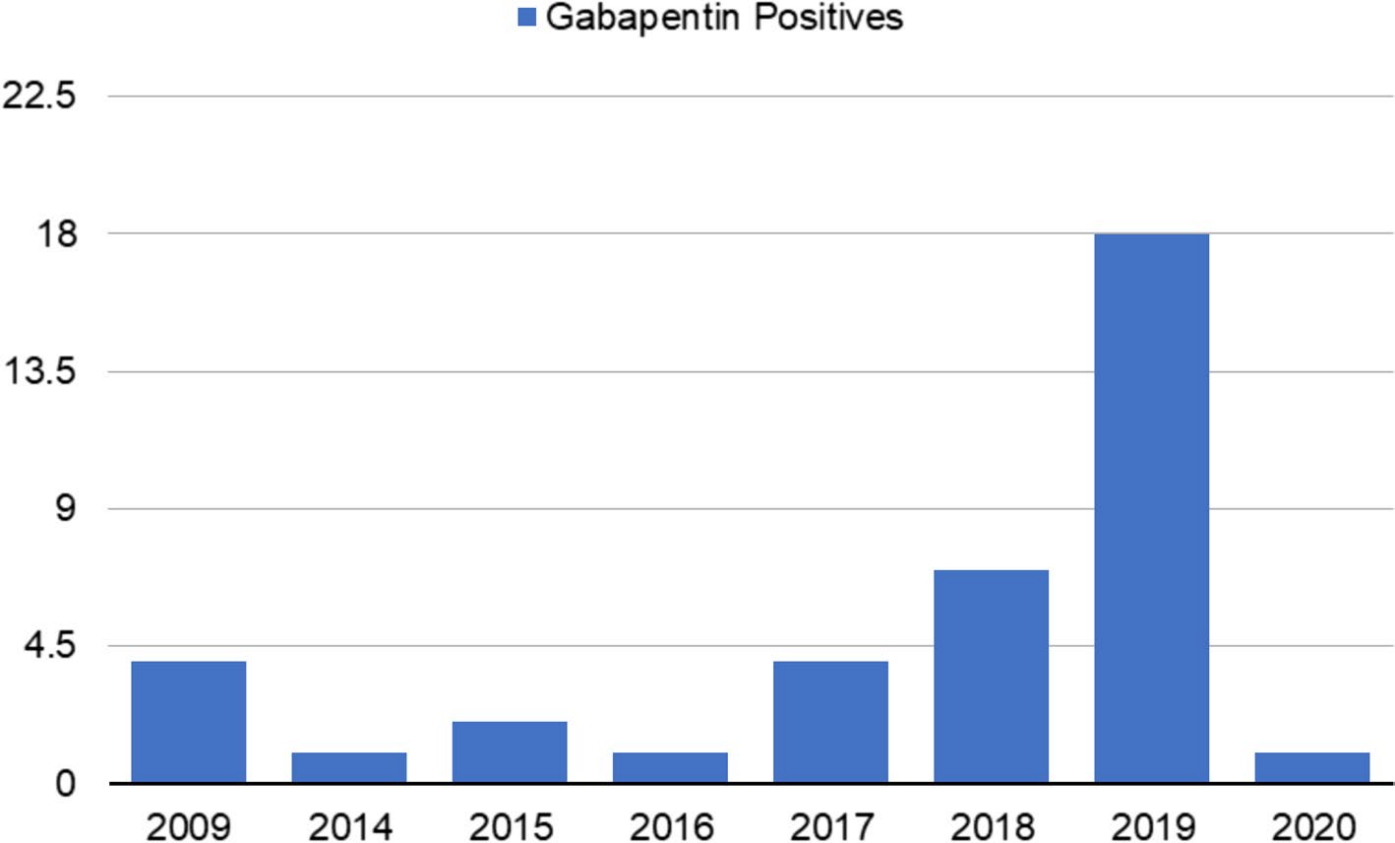
This figure presents annual Gabapentin identifications as per Association of Racing Commissioners International Records, 2009–2020. The year 2019 saw 18 total cases, 8 in Ohio and another 10 called in Ohio but not all of these Ohio cases had been adjudicated by mid-2020

To our knowledge the first Gabapentin *“positive”* of this Ohio sequence came from a race on January 6^th^, 2018, followed by two more, these races on Oct. 23^rd^ and Nov. 12^th^, 2018, for total of three Gabapentin “*positives”*in Ohio racing in 2018 [3]. These Gabapentin “*positives*” resumed in May 2019 and continued for a total of 18 through to July 30^th^, 2020, constituting a significant fraction of the total number of Gabapentin *“positives*” called in US racing, as presented in Fig. 1. In Ohio the basic penalty imposed was purse redistribution, a US$ 500.00 fine, and a 15 day DisQualification (DQ), although on occasion the fine and DQ times were increased. Ohio horsemen were therefore fully aware of these Gabapentin “*positives*” and the associated penalties but were obviously unable to take any action to avoid these low concentration Gabapentin “*positives”* and the resultant penalties.

**Fig. 2:**
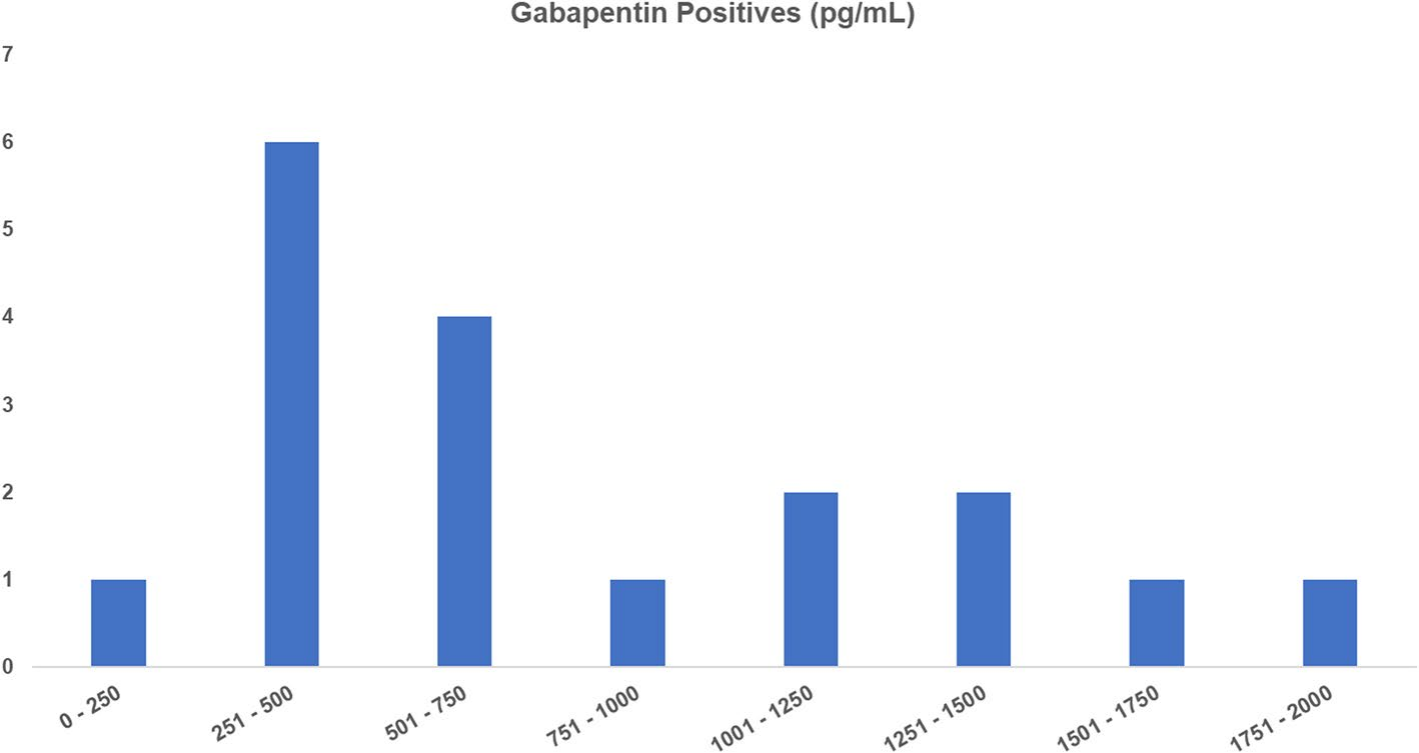
“Estimated” plasma concentrations in pg/ml of the Gabapentin *“positives”* reported in Ohio racing, January 1^st^, 2019, to July 30^th^, 2020, data courtesy of a Freedom of Information (FOI) request to the Ohio Department of Agriculture

The best available quantitative data relating to these Ohio Gabapentin*”positives”* are the estimated concentrations reported present in a sequence of samples called “*positive”* in Ohio from January 1^st^, 2019, to July 30^th^, 2020, as presented in Fig. 2, these data provided under a Freedom Of Information (FOI) request to the Ohio Dept. of Agriculture [4]. These “*estimated”* concentrations range from 230 pg/ml to 1,800 pg/ml, with the majority of the “*positives”* being less than 1 ng/ml in plasma. These ongoing 1 ng/ml or so Gabapentin “*positives”* in equine plasma, continued over a period of approaching 1 year or more in the face of penalties for horsemen, completely consistent with and speaking to the horsemen involved being unaware of the origins of these Gabapentin “*positives”* [3].

The classic example in horse racing of “*positives”* of unknown origin is the sequence of racing chemistry “*positives “*for Aminorex, an amphetamine related substance, many as it happens also in Ohio racing, which were eventually found to be caused by the unexpected metabolic transformation of Levamisole, an equine anthelmintic and immune stimulant, to Aminorex [5]. Identification and communication of the Levamisole origins of these Aminorex “*positives”* led to an immediate reduction in the frequency of these Aminorex *“positives”,* but sporadic *“positives”* continued. More recently Barbarin, an Aminorex related substance present in Brasicaceae pasture plants /weeds, in Kentucky *Barbarea vulgaris,* colloquially “Yellow Rocket “, has been shown to be a plant source of Aminorex “positives”, presumably explaining at least some of the recent Aminorex *“positives”*reported in American racing and also in European sport horses [6].

## Gabapentin as an environmental substance

**Fig. 3:**
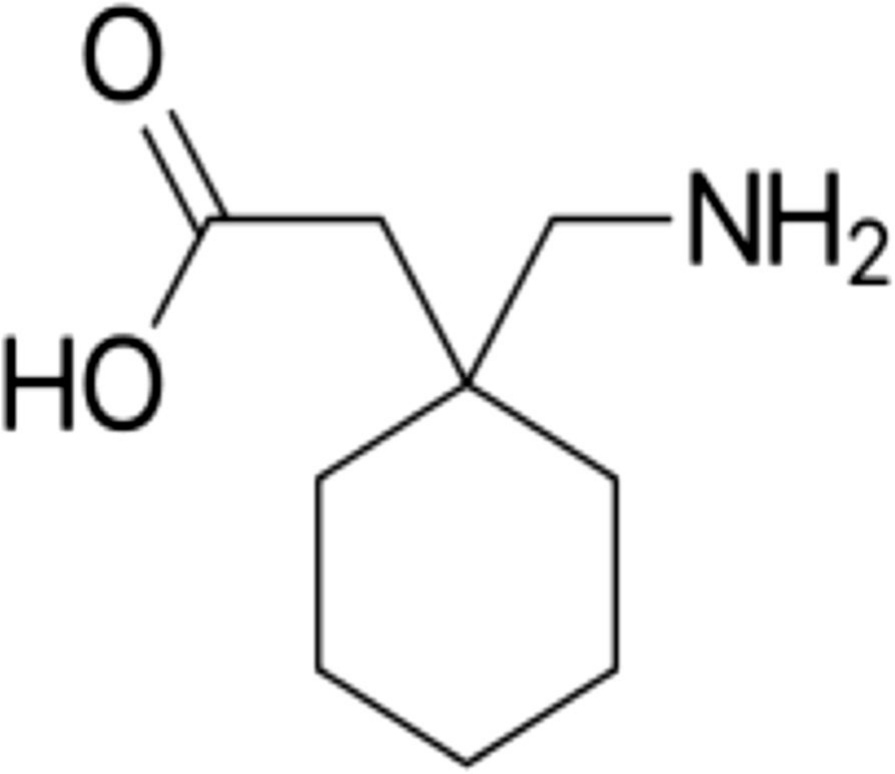
Chemical structure of Gabapentin, 1-(aminomethyl)cyclohexaneacetic acid, C_9_H_17_NO_2,_ molecular weight 171.24

With regard to the origins of these recent low plasma concentration Ohio Gabapentin *“positives*”, environmental Gabapentin resulting from excretion of human prescription Gabapentin is a / the obvious candidate. Gabapentin, Fig. 3 above, is a human anticonvulsant medication used to treat neuropathic pain, hot flashes and restless leg syndrome and a number of other human conditions. Gabapentin is the 11^th^most frequently prescribed medication in the US, it is at times prescribed in combination with opiates, and it is reportedly also used recreationally by humans, presumably at times also in combination with opiates [7]. Gabapentin is also prescribed for similar conditions in veterinary medicine, as we will detail later.

**Fig. 4:**
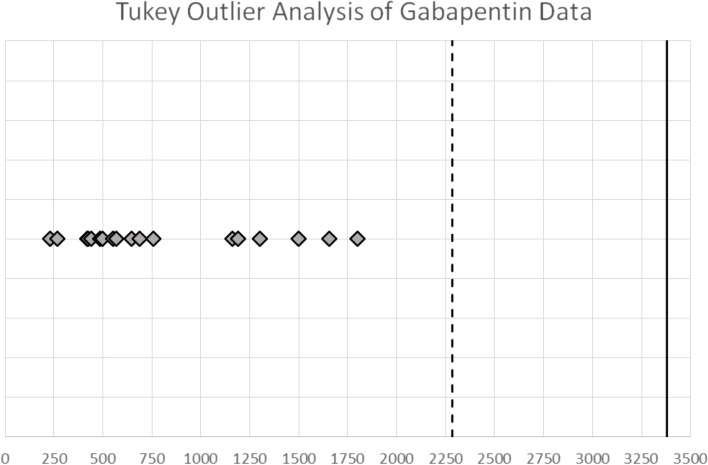
Tukey Outlier Analysis based on the Ohio Department of Agriculture data showing the outlier fences to be 2,282.5 pg/ml (2.3nanograms/ml) for moderate outliers and 3,381.25 pg/ml (3.4 ng/ml) for extreme outliers

With regard to human prescription use of Gabapentin, the prescribing records for Franklin County, Ohio, show that in September of 2019 a total of about 30,000 prescriptions were written for Gabapentin. If we assume an average prescribed dose of 2 g/day, this is 2 million milligrams / day or 2 kg/day prescribed each and every day in Franklin County, the Ohio county in which the Scioto Downs racetrack is located (https://www.ohiopmp.gov/Stats.aspx) [8]. With regard recreational use of Gabapentin, starting in 2017, three Ohio neighboring states, namely Kentucky, Michigan, and West Virginia, and also non-neighboring Tennessee and Alabama, elected to classify Gabapentin as a Drug Enforcement Administration (DEA) Schedule V Controlled Substance, but to our knowledge Gabapentin remains a non-DEA regulated substance in Ohio.

The second factor driving the Ohio environmental presence of Gabapentin is the unusually large daily human dose of Gabapentin, the maximum prescribed daily dose being in the order of 2,400–3,600 mg/day, taken in 3 divided doses, making it one of the larger daily dose medications administered to humans.

The third factor concerning Gabapentin is how it is handled by the human body. Chemically, Gabapentin is an amino acid and orally administered Gabapentin is absorbed from the intestine by the Large neutral Amino acid Transporter (LAT-1) a specific amino acid transporter, following which it distributes throughout the body, distributing primarily in body water. This active transporter uptake system means that small oral doses of environmental contamination Gabapentin are likely to be close to fully absorbed, while with higher doses the fraction absorbed and therefore the relative bioavailability declines [9].

Like all amino acids Gabapentin is a zwitterion, a hybrid molecule containing both acidic, i.e., the negatively charged COOH group and also the positively charged basic NH2 group. Gabapentin is therefore not significantly metabolized by the intracellular drug metabolizing systems that modify drug molecules for excretion and Gabapentin is excreted largely unchanged [9]. This means that individuals taking 2,400 mg/day or more of Gabapentin contribute essentially 2.4 g/day or more of Gabapentin per day to their environment. A further concern is that Gabapentin is chemically stable in the environment and is one of many human pharmaceuticals routinely detected in urban wastewater [10].

Given these circumstances, namely 1/ the high daily dose administered to humans, up to 2.4 or more grams/day, both prescribed and recreational Gabapentin, 2/ the fact that essentially all 2.4 g or more are eliminated unchanged by the human, 3/ that it is chemically stable and persists in the environment and 4/ that it is orally absorbed, it is not surprising that inadvertent transfer of Gabapentin from humans prescribed Gabapentin to horses occurs, as has previously been reported [11].

## Racehorse identifications directly linked to humans prescribed gabapentin

Review of horse racing regulatory records show that a number of equine Gabapentin *“positives”* have been directly linked to humans prescribed Gabapentin. In Charles Town, West Virginia, a horse racing on April 20^th^, 2018, was called “positive” for Gabapentin, 3 ng/ ml in plasma, 86 ng/ml in urine. The horse’s groom was prescribed and taking 2,000 mg /day of Gabapentin [11]. In a second Charles Town matter, on June 8^th^, 2019, the plasma concentration was 16.7 ng/ml, again linked to an employee in contact with the horse taking prescription Gabapentin [11].

Similar incidents have occurred in California. In one matter two racehorses tested “positive” for Gabapentin, horse #1 on April 14^th^, 2019, 9–10 ng/ml in urine and Horse #2, April28^th^, 2109, 5–6 ng/ml in urine. The horse’s groom was prescribed Gabapentin, 400 mg TID, and the groom acknowledged urinating in the stall of horse #1. Horse #1 was claimed in the April 19^th^ race and horse #2 was moved into the horse #1 stall on or about April 14^th^. On April 28^th^ the trainer was notified of the Gabapentin positive in horse #1, on which day horse #2 was racing, and which horse also tested Gabapentin “positive”, reported on May 25^th^, 2019 [12]. Similarly, a July 10^th^, 2020 “positive” in California involving 2 ng/ml of Gabapentin in blood was also linked to an individual working around the horses being prescribed Gabapentin and urinating in the stall in question [12]. Simply put, there are a significant number of cases linking Gabapentin “positives” to individuals prescribed Gabapentin and it is also of interest that in all of these cases the plasma concentrations of Gabapentin were below the Toutain Irrelevant Plasma Concentration (IPC) for Gabapentin, consistent with the “positives” being well below a pharmacologically significant concentration, as we will now set forth [13].

## Human urinary concentrations of gabapentin

Pharmacologically, Gabapentin is a low potency medication, so relatively high plasma concentrations are required for pharmacological effect. In humans, the plasma concentrations required for pharmacological effect are in the order of 2 to 15 ug/ml. Given these relatively high plasma concentrations and the fact that Gabapentin is excreted unchanged, the concentrations of Gabapentin found in human urine can be quite significant, as reported by Heltsley et al. (2011) [14].

Reporting on the concentrations of Gabapentin found in human urine samples, Heltsley et al. (2011) [14] identified a median concentration of 259.8 µg /ml, a mean concentration of 430.9 µg/ml and a high end concentration of 35,345 µg/ml, no less than 35 mg/ml in urine. Given that an average human urinary void volume is about 300 ml, an individual excreting Gabapentin at 35 mg/ml in urine could theoretically contribute approaching 10 g or so of Gabapentin per urine voiding into a horse stall or other equine related environment.

## Irrelvant plasma concentrations (IPC) of gabapentin in horses

The pharmacology of Gabapentin in the horse has been described by Terry et al. (2010) [15]. The dose of Gabapentin used was 20 mg/kg, or 9 g to a 453 kg horse, close to the 10 g or so quantity in the above referenced high concentration single human urine voiding. Following IV administration, the median peak plasma concentration was 73 ug/ml and sedation was observable in all horses out to 150 min post IV administration, at which time the mean plasma concentrations of Gabapentin were well above 10 ug/ml. No effects of Gabapentin on heart rate, rhythm or blood pressure were observed after either Oral or IV administrations.

Using these Terry data and applying the well-established Toutain Irrelevant Plasma Concentration (IPC) model, we can estimate the IPC for Gabapentin in the horse. Calculation of the IPC using the Toutain model, and the Terry data gives an Effective Plasma Concentration (EPC) of about 22 micrograms/ml for Gabapentin in horses. Dividing this concentration by 10 to account for horse to horse variability and then by 50 to ensure no pharmacological effect, one obtains the Toutain IPC of 44 nanograms/ml, we note a somewhat conservative estimate, as discussed by Machin et al., 2018 [16]. We also note that this IPC value is well above the 5 ng/ml in plasma suggested interim Screening Limit of Detection proposed by Brewer et al., [11] and the “in house” 8 ng/ml plasma “Screening Limit” for Gabapentin suggested by Dr. Richard A. Sams in response to a request from the Ohio State Racing Commission [17].

These proposed screening limits are also well supported by a Tukey outlier analysis [16] performed on the Fig. 2 Ohio State Gabapentin data, presented in Fig. 4 below. In this analysis the fences are 2.2 ng/ml for a moderate outlier and 3.3 ng/ ml for an “extreme” outlier, fully consistent with the proposed Screening Limits for Gabapentin in equine plasma referenced above.

This review and analysis shows that unusually large amounts of unchanged Gabapentin are excreted into the environment by humans prescribed Gabapentin and also presumably by humans using it recreationally. In those Gabapentin “*positive “* cases where specific human sources in contact with the horse in question were identified, the concentrations of Gabapentin identified in the plasma and urine samples generally ran above 3 ng/ml. On the other hand, in all of these Ohio January 1^st^, 2019, to July 30^th^, 2020 “*positives”*, where to our knowledge no specific human source has been identified, all of the plasma concentrations are lower, with the highest Ohio *“positive”* concentration being 1.8 ng/ml, the median being 0.5 ng/ml and the concentration in question in the September 7^th^, 2019, Scioto Downs racehorse sample being an extremely modest 0.28 ng/ml. As such, this claimed Ohio Gabapentin *“positive*”, at 0.28 ng/ml of Gabapentin in plasma, is an exceptionally low concentration claimed “*positive* “ identification. This low concentration claimed present in this horse, and all of the similarly Irrelevant Plasma Concentrations claimed identified in the Ohio *“positives*” of Fig. 2 are completely consistent with innocent, inadvertent and essentially unavoidable exposure of these horses to trace level environmental amounts of Gabapentin. Of equal importance, there is no possibility whatsoever of an effect on the outcome of the race in question associated with these low concentration claimed plasma *“positives*”, which interpretation is fully supported by the independent analysis and Screening Limit presented in this matter by the Ohio State Racing Commission expert [17].

As this communication was in final draft a report appeared in The Irish Times [18] detailing a circumstance were Gabapentin transferred from a dog to a racing horse which horse then tested “positive “ for Gabapentin. Gabapentin is used as a therapeutic medication in canine medicine and the dog in this case was a large, 45 kg or so Rhodesian Ridgeback, which had been prescribed Gabapentin for a back injury, presumably at a dose rate comparable to that used in humans. The dog had access to the stable in which the horse was kept, which facts were communicated to the regulatory authority in this matter, the Irish Horseracing Regulatory Board (IHRB). As reported in The Irish Times [18], this possibility was investigated by dosing dogs with Gabapentin and an “*extensive laboratory investigation”* was carried out by the English testing laboratory, LGC, which investigation apparently showed “*sufficient scientific evidence for the IHRB to accept (*the presented*) explanation for the post-race result as likely”.* The investigation concluded that the medication was *“unknowingly administered*” to the horse after *“excretion from the dog in the stable”* a somewhat unexpected but fully understandable sequence of events with respect to the well understood transfer of Gabapentin from humans to horses and now from at least one dog prescribed Gabapentin to a racing horse.

## Conclusions

Gabapentin is a high dose human prescription medication that is also used recreationally by humans. Gabapentin is a DEA class 5 scheduled substance in Kentucky, Michigan, and West Virginia, but not in Ohio. Gabapentin is not metabolized by humans, so the full 3 g/day or so human dose is excreted unchanged into the environment, at times at remarkably high concentrations in human urine. Gabapentin is chemically stable and persists in contaminated environments. As such, inadvertent transfer from humans using Gabapentin to horses occurs, as is clear from the data analyzed and reviewed in this case report.

More importantly, the amounts of environmental Gabapentin transferring to horses are usually minimal and all of these referenced Ohio equine plasma concentrations are an order of magnitude or more below the best available estimates of the conservative Toutain Irrelevant Plasma Concentration (IPC) of Gabapentin in horses, calculated at about 44 ng/ml in plasma. These findings therefore strongly support the proposed Ohio State Racing Commission in place Screening Limit Of Detection (SLOD) of 8 ng/ml for Gabapentin in equine plasma and we note that this Screening Limit Of Detection is actually five-fold more conservative than the itself quite conservative 44 ng/ml Toutain calculated Irrelevant Plasm Concentration (IPC) for Gabapentin in equine plasma.

## Data Availability

The datasets used and/or analyzed during the current study are available in the public domain as referenced in the manuscript or from the corresponding author on reasonable request.

## Abbreviations

IPC: Irrelevant Plasma Concentration
SLOD: Screening Limit of Detection
OHRC: Ohio Horse Racing Commission
ODA: Ohio Department of Agriculture
DQ: DisQualification
FOI: Freedom Of Information
LAT-1: Large Neutral Amino acid Transporter
DEA: Drug Enforcement Administration
EPC: Effective Plasma Concentration
IHRB: Irish Horseracing Regulatory Board
